# Effect of *Cucumis sativus* on Dysfunctional 3T3-L1 Adipocytes

**DOI:** 10.1038/s41598-019-49458-6

**Published:** 2019-09-16

**Authors:** Méndez-Martínez Marisol, Trejo-Moreno Celeste, Maldonado-Mejía Laura, Esquivel-Guadarrama Fernando, Pedraza-Chaverri José, Zamilpa Alejandro, Medina-Campos Omar, Alarcón-Aguilar Francisco, Almanza-Pérez Julio César, Contreras-Nuñez Erika, Santana-Calderón Angélica, Fragoso Gladis, Jiménez-Ferrer Enrique, Rosas Gabriela

**Affiliations:** 1 Instituto de Investigación en Ciencias Básicas y Aplicadas, Universidad Autónoma del Estado de Morelos, Cuernavaca, Morelos, CP 62209 Mexico; 2 Facultad de Medicina, Universidad Autónoma del Estado de Morelos, Cuernavaca, Morelos, CP 62350 Mexico; 3 Laboratorio de Farmacología, Centro de Investigación Biomédica del Sur, Instituto Mexicano del Seguro Social, Xochitepec, Morelo, CP 62790s Mexico; 4 Departamento de Biología, Facultad de Química, Universidad Nacional Autónoma de México, Coyoacán, Ciudad de México, CP 04510 Mexico; 5 Departamento de Ciencias de la Salud, Universidad Autónoma Metropolitana de Iztapalapa, CDMX, C.P 09640 Mexico; 6 Centro de Investigación en Dinámica Celular (IICBA), Universidad Autónoma del Estado de Morelos, Cuernavaca, Morelos, CP 62209 Mexico; 7 Departamento de Inmunología, Instituto de Investigaciones Biomédicas, Universidad Nacional Autónoma de México, Ciudad de México, CP 04510 Mexico

**Keywords:** Medicinal chemistry, Metabolic disorders

## Abstract

Obesity is caused by lipid accumulation in adipose tissues inducing adipocyte dysfunction, characterized by insulin resistance, increased lipolysis, oxidative stress, and inflammation, leading to increased levels of adipokines. Herein the capacity of the subfractions (SFs) SF1, SF2, and SF3 from the *Cucumis sativus* aqueous fraction and their combinations (M) to control adipocyte dysfunction *in vitro*, in 3T3-L1 adipocytes was studied. Adipocytes, previously treated with dexamethasone or IL-1 to induce dysfunction, were incubated with different concentrations of the subfractions for 24 h. 2-deoxyglucose consumption and glycerol release were evaluated, and a surface model was constructed to determine the most effective SF concentrations to improve both parameters. Effective SF combinations were assessed in their capacity to control metabolic, pro-oxidative, and pro-inflammatory conditions. SF1, SF2 (40 μg/ml each) and SF3 (20 μg/ml) improved 2-deoxyglucose consumption by 87%, 57%, and 87%, respectively. SF1 and SF2 (5 μg/ml each) and SF3 (40 μg/mL) increased glycerol secretion by 10.6%, 18.9%, and 11.8%, respectively. Among five combinations tested, only M4 (SF1 40 μg/ml:SF2 60 μg/ml:SF3 30 μg/ml) and M5 (SF1 40 μg/ml:SF2 60 μg/mL:SF3 10 μg/ml) controlled effectively the metabolic, pro-oxidative, and proinflammatory conditions studied. Glycine, asparagine, and arginine were the main components in these SFs.

## Introduction

Obesity is a complex, chronic disease with multifactorial etiology. It is caused by an imbalance between energy consumption and expenditure. Excess energy is stored as fat, which accumulates mainly in adipocytes, increasing their size (hypertrophy) and number (hyperplasia)^1^. Obesity incidence has increased in recent years, becoming a major health problem worldwide. In Mexico, according to the Health and Nutrition National Survey 2016, obesity affects 7 out of 10 Mexican adults (72.5% of adult population)^2^. Obesity has been linked to dyslipidemia, hypertension, glucose intolerance, and insulin resistance (IR), leading to metabolic syndrome^3^. The latter increases the risk of cardiovascular diseases, diabetes mellitus type 2, cancer, and cerebrovascular stroke^4^.

Besides its function as a lipid reservoir, visceral adipose tissue is also an active endocrine organ, producing and secreting various adipokines^5,6^ which do not only modulate adipogenesis, metabolism, and adipocyte function, but also affect appetite and satiety, adipose tissue distribution, insulin secretion and sensitivity, energy release, inflammation, blood pressure, homeostasis, and endothelial function^6^. In obesity, the normal adipokine secretion by adipocytes is affected, altering their homeostasis and inducing dysfunction; lipid and glucose metabolism are first affected, inducing a local IR, which then becomes generalized^7,8^. IR is induced by an alteration of the phosphorylation pathway of the insulin receptor substrate (IRS), in which serine and threonine residues are phosphorylated instead of the usual tyrosine residues; this results in an inhibition of the translocation of the Glucose transporter type 4 (GLUT4) and the induction of glucose intolerance^9–11^. Hyperglycemia, increased levels of free fatty acids and pro-inflammatory cytokines, oxidative stress, and a constant administration of glucocorticoids like dexamethasone are known to induce IR^9,12^.

Adipocyte dysfunction, caused by increased free fatty acid levels, induces an interaction with complexes I and II of the respiratory chain, increasing the cellular concentration of reactive oxygen species (ROS) such as the superoxide anion radical (O_2_•^−^)^13^. In adipose tissues, oxidative stress promotes glucose and lipid oxidation, producing advanced glycation end-products (AGE) and lipid peroxidation end-products (LPE), respectively, which potentiate adipocyte dysfunction and obesity-related disorders^14^. On the other hand, oxidative stress induces the production of IL-1β, TNF-α and IL-6 in adipocytes^1^. These cytokines induce the infiltration of macrophages with a type I phenotype and their activation in adipose tissues, which produce additional IL-1β along with adipocyte-secreted leptin, C-reactive protein (CRP), and resistin. These events potentiate the production of TNF-α and IL-6, inducing a low-grade, chronic inflammation also known as meta-inflammation^5,15,16^. In turn, these inflammation mediators can trigger oxidative stress and exacerbate adipocyte dysfunction^16^.

*Cucumis sativus* is a member of the Cucurbitaceae family; various species of this family have been used to control inflammation, oxidative stress, hyperglycemia, and dyslipidemia^17–22^. In a recent report, the ethanolic fraction of cucumber seed extract was able to reduce total serum lipid levels in a small cohort of adult patients with mild hyperlipidemia^22^. In addition, the *C. sativus* aqueous fraction (Aq-Cs) was found to decrease blood glucose levels in dietary obese mice, improving insulin sensitivity and inducing a regulatory environment in epididymal visceral and subcutaneous adipose tissue (data not published). However, its effect on adipocytes is unknown. The effect of the subfractions (SFs) SF1, SF2, and SF3 of the *C. sativus* aqueous fraction and their combinations on dysfunctional 3T3-L1 adipocytes are reported herein.

IR was induced by dexamethasone and measured as the consumption of 2-deoxyglucose (2-DG) and the release of glycerol. Pro-inflammatory and pro-oxidant conditions were induced by IL-1β and evaluated by IL-6 secretion and oxidative stress induction. Additionally, a surface response model, a mathematical/statistical tool used to analyze the interrelationship among several independent variables and their effect on one or more characteristics of a process^23^, allowed us to determine the most effective subfraction combinations to control these parameters.

## Results

### Aq-Cs SFs did not affect adipocyte viability

**Figure 1:**
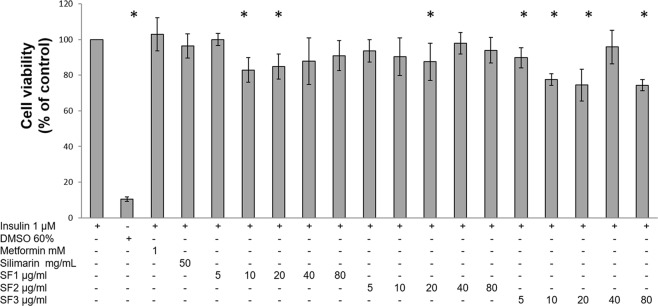
Effect of Aq-Cs subfractions on 3T3-L1 adipocyte cell viability. Cell viability was evaluated by the MTT assay after incubation for 24 h with the three subfractions at a concentration of 5, 10, 20, 40, or 80 μg/mL, and with 60% DMSO. The percentage of viable cells was calculated by defining the viability of untreated cell as 100%. Cells treated with insulin 1 μM were used as a control. Results are expressed as mean ± SD of four independent experiments, *P ≤ 0.05 with respect to insulin-treated cells and were analyzed by ANOVA and the post-hoc Tukey-Kramer test, n = 6.

Determining the effect of all SFs on adipocyte viability was a critical prerequisite to assess their capacity to control insulin resistance induced by dexamethasone and/or pro-inflammatory and pro-oxidant stress induced by IL-1β. As shown in Fig. 1, cell viability ranged from 74.4% to 100% in cultures treated with various SF concentrations (5, 10, 20, 40, and 80 μg/mL), with respect to metabolically healthy adipocytes (adipocytes cultured with insulin 1 μM); in contrast, treatment with 60% DMSO caused a decrease of 89.5% in adipocyte viability with respect to healthy cells (*P* ≤ 0.05).

### Treatment with dexamethasone 1 μM plus insulin 1 μM induced 3T3-L1 adipocyte dysfunction

**Figure 2:**
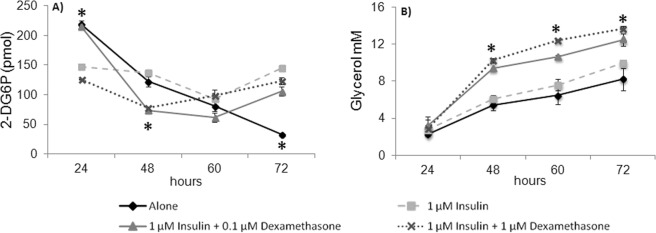
Effect of dexamethasone on mature 3T3-L1 adipocytes. Mature adipocytes (day 8) were cultured for 24, 48, 60, or 72 h, either alone, with insulin 1 μM with or without dexamethasone 0.1 or 1 μM. 2-DG consumption (**A**) and glycerol concentration in culture medium (**B**), were evaluated. Results are expressed as mean ± SD, *P ≤ 0.05 with respect to cells treated with insulin 1 μM and were analyzed by ANOVA and the post-hoc Tukey-Kramer test, n = 6.

The administration of glucocorticoids such as dexamethasone is a well-known cause of IR^12,24^, which is a key trait of adipocyte dysfunction. In adipocytes cultured with dexamethasone, IR is observed as a decrease in glucose consumption and/or the inability to store lipids. As shown in Fig. 2, dexamethasone 1 μM plus insulin failed to alter the consumption of 2-DG (a glucose analog) after 24 h of incubation, while dexamethasone 0.1 μM plus insulin significantly decreased (14.3%, P ≤ 0.05) 2-DG consumption with respect to cells cultured with insulin 1 μM only (Fig. 2A). After 48 h, a decrease (P ≤ 0.05) of 45.8% (0.1 μM) and 43.3% (1 μM) was observed with respect to control cultures. Finally, no significant differences were observed in 2-DG consumption with respect to control cultures after 60 and 72 h of incubation.

Glycerol concentration in the culture medium was another marker of adipocyte dysfunction. No significant differences were observed after 24 h of culture with insulin plus dexamethasone 0.1 μM or 1 μM (*P* > 0.05) with respect to control adipocytes (Fig. 2B). However, a significant increase (P ≤ 0.05) of 54%, 40.1%, and 25.2% was observed in cells treated with dexamethasone 0.1 μM plus insulin after 48, 60, and 72 h of culture, respectively. Glycerol release was increased by 67.8%, 63.4%, and 37.4% in adipocytes treated with dexamethasone 1 μM plus insulin compared to controls after 48, 60, and 72 h, respectively.

### IL-1β induced an increase in IL-6 secretion and ROS production

**Figure 3:**
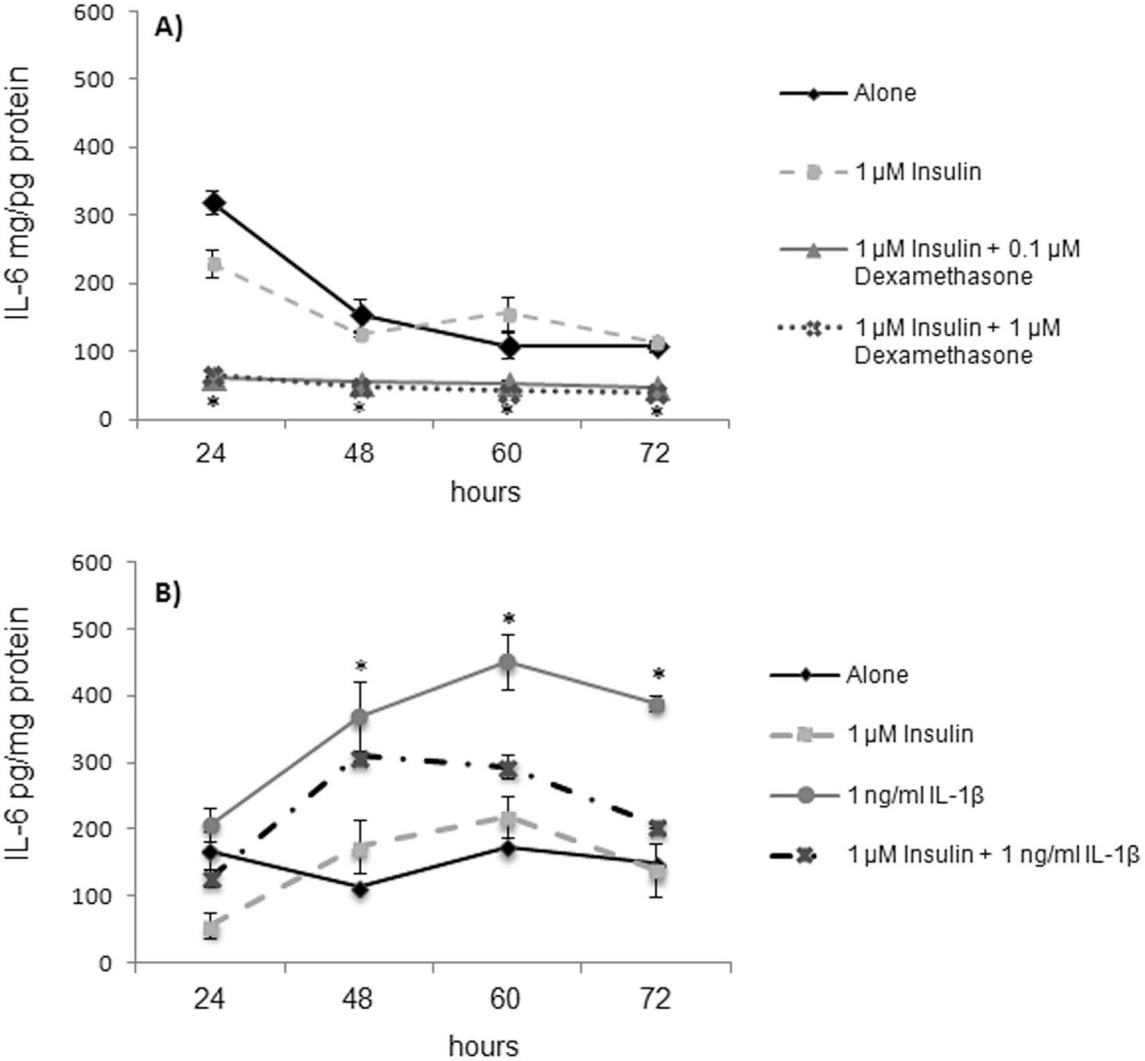
IL-6 (pg/mg protein) concentration in culture medium. Cells were left untreated or treated either with dexamethasone 0.1 or 1 μM (**A**), or with 1 ng/mL of IL-1β (**B**), with or without insulin. IL-6 (pg/mg protein) concentration was determined in culture medium at 24, 48, 60, and 72 h of culture. Results are expressed as mean ± SD, *P ≤ 0.05 with respect to cells treated with insulin 1 μM and were analyzed by ANOVA and the post-hoc Tukey-Kramer test, n = 6.

Considering the capacity of dexamethasone to induce endothelial dysfunction, its capacity to induce a pro-inflammatory status and oxidative stress was assessed. However, it failed to induce a significant increase in IL-6 secretion with respect to control adipocytes at the times evaluated (Fig. 3A). A similar result was obtained in the quantification of O_2_•^−^ (data not shown).

On the other hand, a significant, almost two-fold increase (P ≤ 0.05) in IL-6 secretion with respect to healthy cells was observed when adipocytes were cultured with IL-1β alone for 24 h (Fig. 3B). When insulin (1 μM) was added, a nearly 3-fold increase in IL-6 levels was observed. A similar effect was observed after 48, 60, and 72 h (2.1-/1.7-, 2-/1.3-, and 2.8-/1.4-fold increases, respectively).

**Figure 4:**
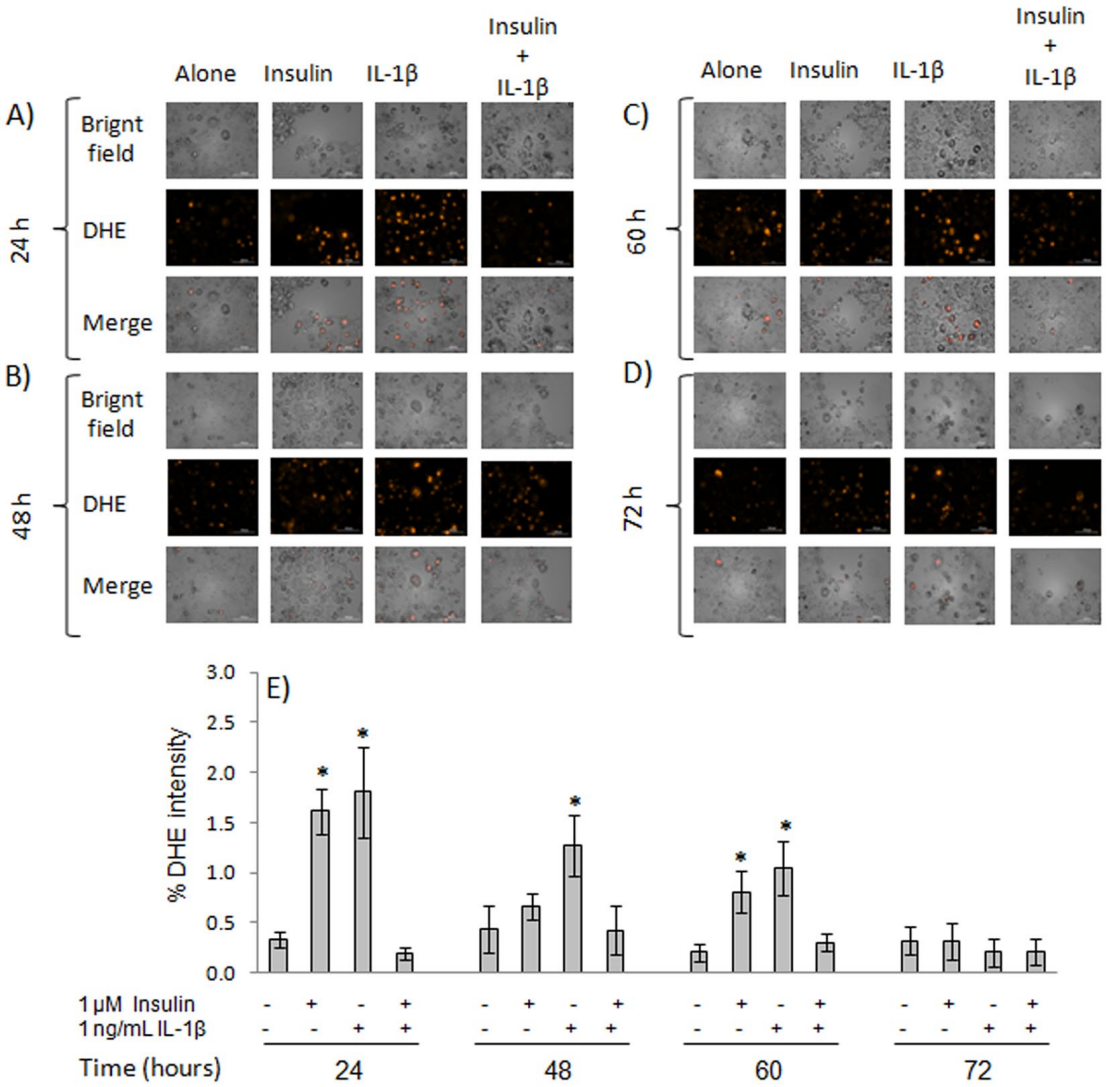
Superoxide induction by IL-1β in 3T3-L1 adipocytes. Mature adipocytes (day 8) were cultured for 24, 48, 60, or 72 h, either alone, with IL-1β, and/or insulin 1 μM. Microphotographs were taken in a spectrofluorometer under a 20X objective at 24 (**A**), 48 (**B**), 60 (**C**), and 72 h (**D**). The percent of DHE expression was determined (**E**) with the software MetaMorph v.6.1. Results are expressed as mean ± SD, *P ≤ 0.05 with respect to cells treated with insulin 1 μM and were analyzed by ANOVA and the post-hoc Tukey-Kramer test, n = 6.

As shown in Fig. 4A,E, a significant increase (P ≤ 0.05) in DHE intensity (about 50%) was observed in adipocytes treated with insulin or with IL-1β for 24 h, indicating higher O_2_•^−^ levels. On the other hand, treatment with IL-1β for 48 h caused an increase of about 50% (P ≤ 0.05) in DHE intensity with respect to insulin-treated cells (Fig. 4B,E). After 60 h of culture, a similar increase of about 50% (P ≤ 0.05) in DHE intensity was observed in cells treated with insulin only, and also in those treated with IL-1β (Fig. 4C,E).

These results indicate that IL-6 secretion and ROS production were increased in adipocytes treated with 1 ng/mL of IL-1β for 48 h. Therefore, this condition was selected to evaluate the capacity of SF mixtures to control these parameters.

### Aq-Cs fractions reverse adipocyte dysfunction

Adipocyte dysfunction was induced by culturing mature adipocytes with dexamethasone 1 μM and insulin 1 μM for 48 h. The effect of Aq-Cs SF1, SF2, and SF3 was evaluated by measuring glucose consumption and glycerol release compared to silymarin- and metformin-treated cells.

**Figure 5:**
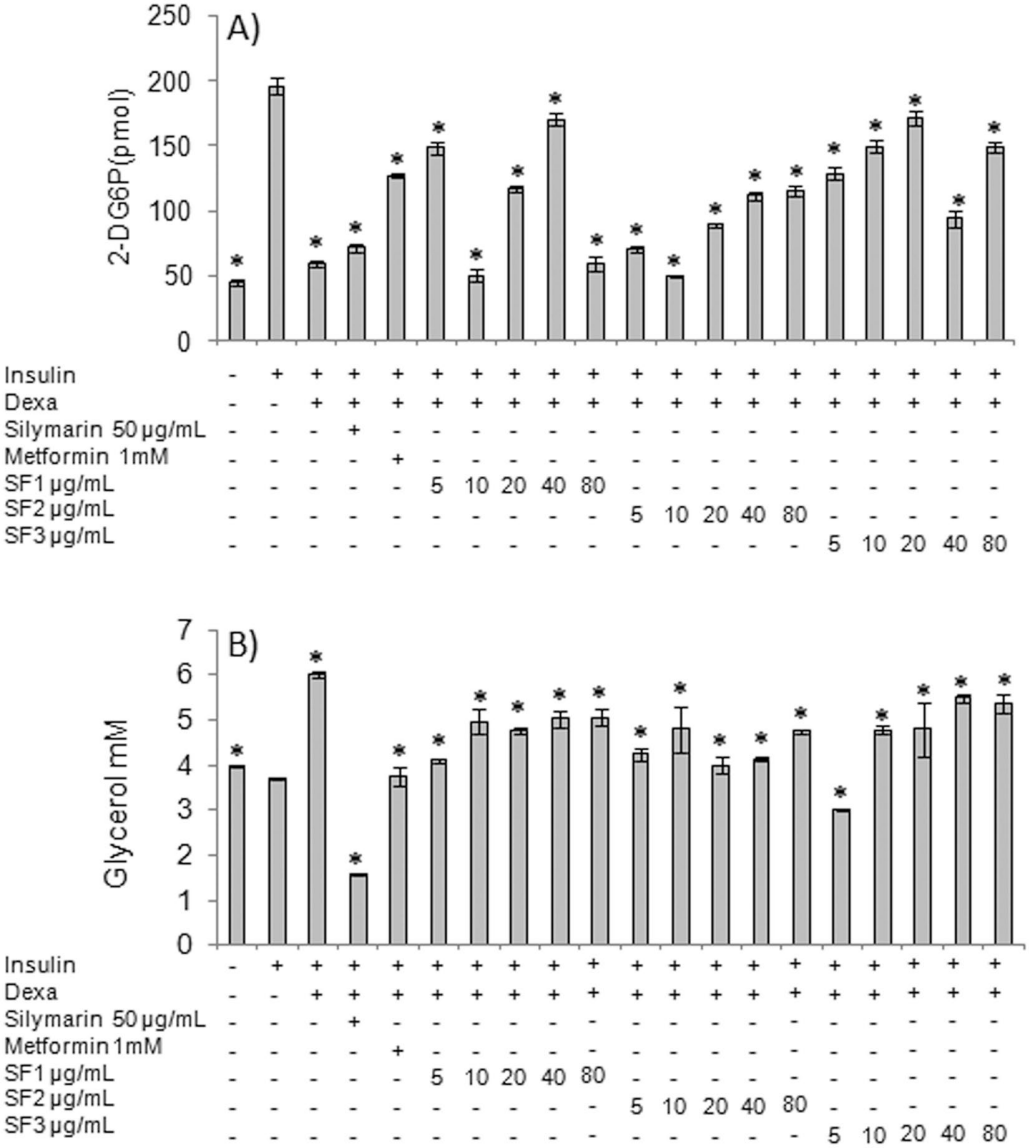
Effect of SF1, SF2, and SF3 Aq-Cs subfractions on 2-DG consumption and glycerol release. Mature adipocytes were cultured for 48 h with dexamethasone 1 μM plus insulin 1 μM. Then, the subfractions SF1, SF2, and SF3 were added at a concentration of 5, 10, 20, 40, or 80 μg/mL and incubated for a further 24 h period. The effect of the subfractions on 2-DG consumption (**A**) and glycerol concentration in culture medium (**B**) was evaluated. A response surface model was constructed to identify optimal combinations of subfractions at an effective concentration for 2-DG consumption (**C**) and glycerol release (**D**). Metformin and silymarin were used as controls. Results are expressed as mean ± SD, *P ≤ 0.05 with respect to cells treated with insulin 1 μM and were analyzed by ANOVA and the post-hoc Tukey-Kramer test, n = 6.

As shown in Fig. 5A, dexamethasone decreased 2-DG consumption by 70% compared to controls (P ≤ 0.05). Silymarin failed to induce a significant increase in 2-DG consumption, but metformin allowed adipocytes to consume 65% more 2-DG, a significant difference with respect to dexamethasone-treated cells (P ≤ 0.05). On the other hand, SF1 and SF3 induced 2-DG consumption, showing a bimodal effect; peak responses occurred when adipocytes were treated with 40 μg/mL of SF1 and 20 μg/mL of SF3. On the other hand, 2-DG consumption increased as the concentration of SF2 increased, being 40 μg/mL and 80 μg/mL the most effective concentrations, leading to an increase of 57% and 58.9%, respectively.

With respect to glycerol levels in culture media (Fig. 5B), adipocytes treated with insulin 1 μM only released 3.7 mM of glycerol, while those treated with dexamethasone and insulin released 6 mM, a two-fold increase (P ≤ 0.05) compared to control cells. On the other hand, a significant decrease in glycerol concentration (1.5 mM, P ≤ 0.05) was observed in silymarin-treated cells; metformin-treated cells showed glycerol levels similar to healthy cells (P > 0.05).

SF1 and SF2 increased the release of glycerol in a dose-dependent manner. The most effective concentration of both SF1 and SF3 was 5 μg/mL, while a bimodal response was observed for SF2, for which a concentration of 40 μg/mL kept glycerol concentrations near to the levels observed in controls (*P* > 0.05).

### 2-DG consumption and glycerol release response surface model

To find the most effective mixtures of the selected SFs to regulate 2-DG consumption and glycerol release, a response surface model was constructed. The results of such model are shown in Table 1, and the combinations selected are shown in Table 2. The values obtained are comparable to those observed in controls (silymarin and metformin), >120 pmol/dL for 2-DG consumption, and 1–4 mM for glycerol release.

**Table 1:** Experimental results of the factorial fractional design.

Treatment	SF1	SF2	SF3	2-DG consumption	Glycerol release
	*x*_1_	*x*_2_	*x*_3_	*Y* (pmol/dL)	*Y* (mM)
1*	−1	−1	−1	149.5	2.1
2*	−1	−1	0	126.5	1.8
3*	−1	−1	1	162.4	1.5
4^#^	0	1	−1	194.9	1.6
5^#^	0	1	1	160.2	1.5

^*^1,2,3: *x*_1_ = ((*X*_1_ − 5)/2.5), *x*_2_ = ((*X*_2_ − 40)/20), *x*_3_ = ((*X*_3_ − 5)/2.5).

^#^4,5: *x*_1_ = ((*X*_1_ − 40)/20), *x*_2_ = ((*X*_2_ − 40)/20), *x*_3_ = ((*X*_3_ − 20)/10).

**Table 2:** Effective combinations according to the response surface model for 2-DG consumption and glycerol release.

Combination	SF1 (μg/mL)	SF2 (μg/mL)	SF3 (μg/mL)
M1	2.5	20	7.5
M2	2.5	20	2.5
M3	2.5	20	5.0
M4	40	60	30
M5	40	60	10

**Figure 6:**
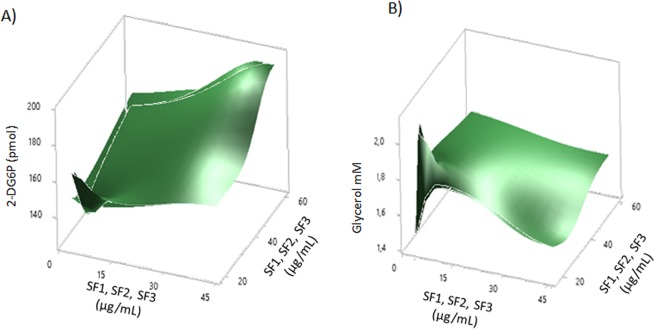
Response surface plot. A response surface model was constructed to identify optimal combinations of fractions at an effective concentration. For this, combinations of the effective fractions were added to dysfunctional 3T3-L1 adipocytes and incubated for an additional 24 h. The effect of the combinations on the consumption of 2-DG (**A**) and the release of glycerol (**B**) was evaluated. The plot shows the effect of combinations of the effective fractions for both parameters.

Three-dimensional plots were obtained by calculating the surface response for 2-DG consumption and glycerol release (Fig. 6A,B). As shown, the combinations causing a peak in 2-DG consumption were M4 and M5 (Fig. 6A); these combinations kept glycerol levels in a range of 1–2 nM at the lower region of the surface model (Fig. 6B).

### Combinations M4 and M5 reverse dysfunction induced by IL-1β

To determine whether the effective subfractions exhibit anti-inflammatory and anti-oxidant activity when administered together, the five combinations obtained from the response surface model were experimentally evaluated on IL-1β-induced dysfunctional adipocytes.

**Figure 7:**
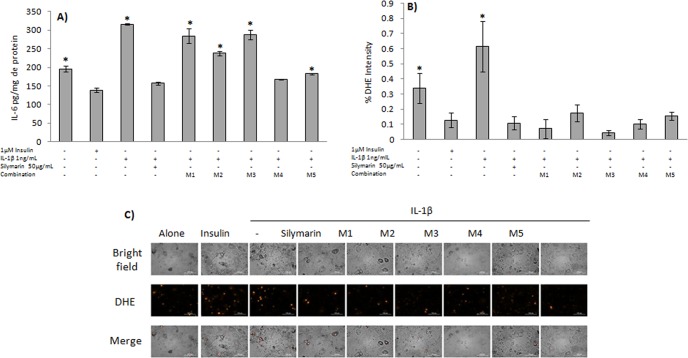
Effect of the combinations of effective subfractions on IL-6 production and O_2_•^−^ production. Mature adipocytes were cultured for 48 h either with 1 ng/mL of IL-1β or with insulin 1 μM. Then, the combinations M1, M2, M3, M4, and M5 of the effective fractions were added and incubated for a further 24 h period. The effect of the combinations assayed on IL-6 concentration (**A**), and O_2_•^−^ levels (**B**,**C**) were evaluated. Metformin and Silymarin were used as controls. Results are expressed as mean ± SD. Microphotographs were taken in a spectrofluorometer under a 20X objective. *P ≤ 0.05 with respect to cells treated with insulin 1 μM and were analyzed by ANOVA and the post-hoc Tukey-Kramer test, n = 6.

Treating adipocytes with IL-1β induced a significant increase (by almost 50%, Fig. 7A) in IL-6 concentration with respect to healthy adipocytes (P ≤ 0.05). The combinations M4 and M5, on the other hand, kept IL-6 concentrations close to the values observed in insulin-treated and silymarin-treated dysfunctional adipocytes (P > 0.05).

To evaluate the anti-oxidant capacity of the effective mixtures of Aq-Cs SFs, O_2_•^−^ levels were determined in dysfunctional adipocyte cultures. As shown in Fig. 7B,C, adipocyte treatment with IL-1β caused a five-fold increase in O_2_•^−^ levels with respect to insulin-treated adipocytes (P ≤ 0.05). Silymarin, a flavonoid with anti-oxidant activity^25^, decreased O_2_•^−^ production by 82.5% (P ≤ 0.05). The combinations M4 and M5 showed a similar effect to that of silymarin; M1 and M3 decreased O_2_•^−^ production by 88.6% and 92.7%, respectively, compared to IL-1β-treated adipocytes, although no significant differences compared to silymarin nor the combinations M4 and M5 were observed (P > 0.05). These results indicate that the compounds in the mixtures M4 and M5 have both a significant anti-oxidant and anti-inflammatory capacity, similar to that of silymarin.

### SF1, SF2, and SF3 contain amino acids

To identify the components in SFs that could be responsible for the observed biologic effects, thin-layer chromatography and mass spectrometry analyses with various amino acid standards were performed. The results (Supplementary Figs 1 and 2) indicate that amino acids are the major components of all subfractions. Positive ionization yielded few peaks with molecular weight of 58.96, 128.93, and 157.97 Da, corresponding to glycine, lysine, and arginine, respectively; negative ionization yielded a peak with molecular weight of 113.00 Da, which corresponds to asparagine (Table 3).

**Table 3:** Amino acids identified in the subfractions from Aq-Cs by mass spectrometry.

Ion mode	Precursor peak (m/Z)	Amino acid	Molecular formula
Positive	58.96	Glycine	C_2_H_5_NO_2_
Negative	113.00	Asparagine	C_4_H_8_N_2_O_3_
Positive	128.93	Lysine	C_6_H_14_N_2_O_2_
Positive	157.97	Arginine	C_6_H_14_N_4_O_2_
Positive	105.93	Serine	C_3_H_7_NO_3_
Positive	73.84	Alanine	C_3_H_7_NO_2_
Positive	117.96	Valine	C_5_H_11_NO_2_
Positive	132.00	Leucine	C_6_H_13_NO_2_
Positive	133.96	Aspartic acid	C_4_H_7_NO_4_
Positive	147.96	Glutamic acid	C_5_H_9_NO_4_

## Discussion

Recently it was demonstrated that aqueous soluble compounds from aerial parts of *Cucumis sativus* down regulated the inflammatory and oxidative effects induced by Ang II in human endothelial cells^26^. In this study a similar process of elimination of less polar compound (ethyl acetate fraction) was used to obtain fractions SF1, SF2 and SF3. The effect theses mixtures of metabolites obtained from the Aq-Cs fraction, as well as of their combinations (M1-M5) was assessed on 3T3-L1 adipocytes (cell line which once differentiated to adipocytes *in vitro* can be used as a model of this cell type) in which dysfunction was previously induced by culturing them in the presence of either dexamethasone or IL-1β. Adipocyte dysfunction and IR were evaluated by the production of IL-6 and ROS like O_2_•^−^ ^13^, as well as glucose consumption (measured as 2-DG)^7^, and the production and release of free fatty acids (measured as glycerol levels in culture medium)^9^.

Two IR inducers were used in this study, dexamethasone and IL-1β. Dexamethasone induces IR by altering the insulin signaling pathway by inhibiting phosphatidylinositol-3-kinase (PI3K) and serine/threonine protein kinase (Akt), thus preventing the translocation of GLUT4 to the cell surface, which leads to glucose intolerance (a decrease in its consumption)^27,28^. On the other hand, dexamethasone also increases the activity of the hormone-sensitive lipase (HSL), which results in increased cell lipolysis and therefore in an increased release of glycerol and free fatty acids by adipocytes into the medium^29,30^. Dysfunctional adipocytes were used to evaluate the effect of the SFs and their combinations on 2-DG consumption and glycerol release (Fig. 2A,B).

In addition, being dexamethasone a synthetic glucocorticoid, acts as an anti-inflammatory, preventing the expression of IL-6 by binding and activating glucocorticoid receptors, which in turn bind specific NF-κB-binding sites in DNA, recruiting cofactors and modifying chromatin to prevent the expression of pro-inflammatory cytokines^31^; thus, in contrast with IL-1β, dexamethasone failed to increase IL-6 production (Fig. 3A).

On the other hand, inflammation and oxidant stress are closely related to IR, potentiating the dysfunctional status of adipocytes^32^. To induce both conditions, the cells were treated with IL-1β (Figs 3B and 4A–E), which: (1) Induces stress in the endoplasmic reticulum, leading to an uncoupling protein response (UPR), which in turn leads to the production of ROS^33^; (2) Increases the production of O_2_•^−^ by mitochondria through a reduction catalyzed by cytochrome oxidase^34^; (3) Activates the c-Jun N-terminal kinase (JNK) and IKK (an NF-κB inhibitor), thus favoring the pro-inflammatory status and increasing the secretion of cytokines like IL-6^31^. IL-6 is regarded as a marker of adipocyte dysfunction, since it indicates a pro-inflammatory status of the cell^35^, and it is even a marker of adipocyte damage^15,16^. By itself, this interleukin potentiates cell dysfunction by increasing lipolysis and induces glucose intolerance in 3T3-L1 adipocytes^36,37^, favoring IR. This is in contrast with dexamethasone, which due to its anti-inflammatory activity does not modify IL-6 nor O_2_•^−^ levels.

The three subfractions reversed efficiently dexamethasone-induced IR at the different concentrations evaluated, with no toxic effects for the cells (Fig. 1). One of the most abundant components in all SFs was glycine (Supplementary Fig. 2); this amino acid has been reported to favor glucose consumption by glucose-intolerant adipocytes, since it helps GLUT4 to reach the cell surface and perform glucose transport. Previous reports indicate that glycine exerts these effects because it: (1) Acts in a similar manner as metformin, increasing the activity of AMP-activated protein kinase (AMPK) and therefore the expression of GLUT4^37,38^; (2) Induces the expression of GLUT4 through PPARγ activation and adiponectin secretion^39^. On the other hand, glycine has also been reported to negatively modulate the expression and activation of lipoprotein lipase (LPL) and LSH^40^, thus controlling lipolysis and the release of glycerol into the medium.

Another amino acid found in all three SFs is arginine, which is known to regulate lipolysis; arginine is the substrate for nitric oxide synthase (NOS) to produce nitric oxide (NO), which increases the expression of the PPAR-γ coactivator 1-alpha, which in turn regulates oxidative phosphorylation. On the other hand, ON regulates the expression of ROS (like O_2_•^−^) and AMPK, inducing glucose and lipid oxidation in adipose tissue and in liver and muscle^41,42^. Regarding the asparagine, the other amino acid found in the SFs, no reports have been found to date on anti-hyperglycemic, anti-inflammatory, or anti-lipidemic activity of asparagine, thus further studies using this amino acid alone would be convenient to perform in order to evaluate its properties in the above pathologies of the endothelial dysfunction. Since all three SFs were efficacious (although in differing concentration levels) to control glucose intolerance and lipolysis (Fig. 5A,B), a response surface model was used to determine the best SF combinations. This model is based on the analysis of the relationship between independent variables measuring one or more characteristics of a process^43^; and it allowed us to select five combinations (M1-M5), which proved to induce a better control on 2-DG consumption and lower glycerol release (Table 1), being this effect even more efficient than with any SF alone (Fig. 5A,B).

These combinations were evaluated in their anti-oxidant and anti-inflammatory capacity; according to our results, two combinations (M4 and M5) exhibited anti-inflammatory effects (decreased IL-6 levels, Fig. 7A) and anti-oxidant effects (decreased O_2_•^−^ levels) similar to those of silymarin, a flavonoid with well-documented anti-oxidant and anti-inflammatory activity^44–46^.

Our results indicate that combinations M4 and M5 not only were efficient to control inflammation and oxidant stress, but also to increase the consumption of 2-DG and decrease glycerol release. Therefore, these combinations could control the IR that accompanies obesity, because it is related with the presence of oxidant stress and the secretion of IL-6^35,36^. Glycine and arginine have been reported to decrease the expression of IL-6 and TNF-α mRNA, and therefore they have anti-inflammatory effects^24^. Additionally, glycine acts as an anti-oxidant by increasing the synthesis of glutathione, thus protecting cells from OS^47^. Arginine also plays a key role in regulating OS, decreasing the levels of ROS and increasing the levels of anti-oxidant enzymes like superoxide dismutase (SOD), glutathione peroxidase (GPX), and CAT in dysfunctional adipocytes^25^. Both amino acids increase the levels of copper/zinc-superoxide dismutase (Cu/Zn-SOD), an anti-oxidant enzyme capable of reducing superoxide ion to hydrogen peroxide^48^.

Considering that not all combinations were equally efficient to modulate dysfunctional adipocytes, the proportion of their constituents is relevant. Thus, it is noteworthy that M4 and M5 were the combinations with the highest concentration of the tree subfractions, indicating the possible usefulness of the plant extract to control pathologies linked to metabolic syndrome.

## Conclusion

We showed that subfractions SF1, SF2, and SF3 from the *Cucumis sativus* aqueous extract and their combinations M4 and M5 are effective to control dysfunction in 3T3-L1 adipocytes, induced either by dexamethasone or IL-1β. These two combinations proved to be promising candidates for an alternative treatment with anti-oxidant and anti-inflammatory effects, capable of inducing insulin sensitivity and regulate lipid metabolism.

## Materials and Methods

### Chemicals and reagents

Standards rutin, quercetin, glucose, valine, proline, glycine, arginine, leucine, asparagine, lysine, isoleucine, aspartate, and glutamate; reagents Naphthol, and aminoethanol dimethylborate, 3-isobutyl- 1-methylxanthine (IBMX), dimethyl sulfoxide (DMSO), dexamethasone, insulin, 3-(4,5-Dimethylthiazol-2-yl)-2,5-diphenyl- tetrazoliumbromide (MTT), dihydroethidium (DHE), silimaryn, where obtained from Sigma-Aldrich (St. Louis, MO, USA). High-glucose Dulbecco’s Modified Eagle’s Medium (DMEM) and calf bovine serum were purchased from ATCC (Rockville, MD, USA). Reagents 4-hydroxybenzaldehyde and ninhydrin was from Merck Millipore (Burlington, MA, USA). The pierce Coomassie Bradford Protein Assay Kit, Fetal bovine serum (FBS), Gentamicin was obtained Thermo Fisher Scientific (Waltham, MA, USA). Reagents dichloromethane, methanol, ethyl acetate, glacial acetic acid, n-butanol, acetone, acetonitrile, sulfuric acid (H_2_SO_4_) were purchased from J.T. Baker (Madrid).

#### Plant material and *Cucumis sativus* fractions

*Cucumis sativus* aerial parts, including leaves, stems, and fruits were collected from a pesticide- and fertilizer-free edible crop in Xochitepec, Morelos, Mexico, in July-August. Plant material was identified by Biol. Margarita Avilés Flores and Macrina Fuentes Mata (Herbario del Jardín Etnobotánico del Instituto Nacional de Antropología e Historia, INAH, Morelos city). Voucher specimens were stored at this site for future reference (INAH-Morelos 3001). The plant material was kept away from light at room temperature (RT) and dried in an oven at 50 °C for 36 h, following a procedure previously described^26^. Extracts were obtained immediately after the material was completely dried. Dried plant material was ground in an electric mill to a particle size of 4 mm. This powder (1.12 kg) was extracted by a maceration process with an ethanol/water (60:40) solution at RT. The liquid extract was paper-filtered, concentrated in a rotary evaporator Laborota 4000 (Heidolph, Germany) under reduced pressure at 50 °C. The semisolid extract was finally freeze-dried to obtain 153 g of a dry green powder. The crude extract (50 g) was diluted in distilled water (500 mL) and partitioned with ethyl acetate (500 mL) in a separation funnel. The organic phase was discarded, and the volume of the aqueous fraction was reduced by low-pressure distillation to obtain a semisolid sample, which was finally freeze-dried to obtain 140 g of a green powder. This mixture (Aq-Cs, 35 g) was suspended in acetone (700 mL) for 24 h. Upon filtration, the soluble phase was concentrated and the solvent was completely removed by a distillation process under reduced pressure followed by high vacuum drying, in a rotary evaporator to obtain the subfraction SF1 (3.2 g). The precipitate was resuspended in methanol (700 mL), obtaining a solution, which was then concentrated by low pressure distillation (subfraction SF2, 4.7 g), and a precipitate (subfraction SF3, 13.3 g).

### Thin-layer chromatography

Chemical profile of subfractions SF1, SF2 and SF3, was performed using a thin-layer chromatography (TLC) assay which was developed both in normal- and in reverse-phase, following a procedure previously described^26^. Commercially available standards rutin, quercetin, glucose, valine, proline, glycine, arginine and leucine were used as references. Mixture of dichloromethane:methanol (7:3 v/v), ethyl acetate:methanol:water:glacial acetic acid (7:2:2:0.5 v/v), n-butanol:acetone:glacial acetic acid:water (35:35:10:20 and 70:70:20:10 v/v) and water:acetonitrile (7:3 v/v) were used as mobile phases. Once the plates were developed, spots were visualized with Naphthol for polysaccharides, 4-hydroxybenzaldehyde for flavonoids, aminoethanol dimethylborate for flavonoids, or ninhydrin for amino acids, following the manufacturer’s instructions. The development was carried out on exposure of the plates to UV light (254 nm).

#### ULPC-MS Analysis

To identify the bioactive compounds in SF1, SF2 and SF3, all three SFs were subjected to chromatographic analysis using an Acquity UPLC system (Waters, Milford, MA, USA), equipped with a quaternary pump and autosampler column oven. Liquid chromatography was performed at 30 °C, using an Acquity UPLC BEH 1.7 C18 column (2.1 × 10 mm × 1.7 mm i.d.). The column was eluted with a gradient system consisting of 0.1% formic acid in water (A) and 0.1% formic acid in acetonitrile (B) at a flow rate of 0.3 mL/min. Mobile phase gradient was set at 100% A for 2 min and subsequently ramped to 100% B (curve 6) over 14 min, followed by a 1 min at 100% B before a rapid return to 100% A, and an equilibration period of 2 min. Run-to-run time was 20 min. Volume of injection was 5 µL. Mass spectrometry analysis was performed and analyzed in a triple quadrupole TQD mass spectrometer (Waters) through an electrospray Z-spray ion source, in ESI-positive mode. Source and desolvation temperatures were 150 and 450 °C, respectively. A combination of cone voltage of 20 V and capillary voltage of 2.5 kV was used. Nitrogen was employed both as desolvation gas and cone gas. Optimal detection conditions were determined by constant infusion of standard solutions (50 µM) in solvent. MS scan was performed using argon as the collision gas. Detection conditions were determined by constant infusion of standard solutions (50 µg/mL) in acidulated water (0.05% trifluoracetic acid). To identify the major amino acids contained in each fraction, commercial standards of glycine, arginine, asparagine, lysine, leucine, isoleucine, aspartate, and glutamate were used as chromatographic standards.

#### Cell culture

Murine 3T3-L1 fibroblasts were purchased from the American Type Culture Collection (ATCC CL-173; Rockville, MD, USA) and cultured in high-glucose Dulbecco’s Modified Eagle’s Medium (DMEM) supplemented with 10% calf bovine serum, 20 μg/mL of gentamicin and incubated at 37 °C, 5% CO_2_, for 48 h. At day two post-confluence, fibroblasts were differentiated into adipocytes with dexamethasone 1.0 μM, methylisobutylxanthine 0.5 mM, and 1.0 μg/mL of insulin in DMEM plus 10% fetal bovine serum (FBS). Two days later, the medium was replaced by fresh DMEM plus 10% FBS and 1.0 μg/mL of insulin, and the cells were allowed to mature for 6 days. The cells were processed when 95% of them showed typical adipocyte traits (day 8)^49^.

Once adipocyte dysfunction was established as described below, five concentrations (5, 10, 20, 40, or 80 μg/mL) of the three Aq-Cs SFs (SF1, SF2, and SF3) were added to the culture and allowed to stand for 24 h. Either silymarin (50 μg/mL)^50,51^ or metformin (1 mM)^52,53^ were used as positive controls; previous reports have employed these molecules as standard drug: for silymarin as control of oxidative stress and the inflammatory condition induced by the presence of IL-1β^45,46^. For Metformin it was used as control of glucose consumption and the accumulation of lipids in adipocytes, functions that were affected by the presence of dexamethasone^27–30^.

For cell viability and 2-deoxyglucose (2-DG) consumption assays, 1.5 × 10^3^ adipocytes/well were seeded in 96-well plates. To assess glycerol release, IL-6 concentration, and O_2_•^−^ presence, 4.5 × 10^4^ cells/well were cultured in 12-well plates and further processed (see below).

#### MTT assay

To assess the effect of Aq-Cs SFs on cell viability^4^, mature adipocytes were incubated for 24 h in the presence of different concentrations of the three Aq-Cs SFs (5, 10, 20, 40, or 80 μg/mL) diluted in DMEM plus 10% FBS and insulin (1.0 μg/mL). Additionally, silymarin (50 μg/mL)^50,51^, metformin (1 nM)^52,53^, or DMSO (60%) were used as controls. After 24 h, the cells were incubated with 5 mg/mL of 3-(4,5-dimethylthiazol-2-yl)-2,5-diphenyltetrazolium (MTT) at 37 C for 4 h. After incubation, 100 μL of 10% SDS-HCl 0.01 N (1:1) were added to each well, and the plates were incubated for 2 h at 37 °C. Absorbance was measured at 570 nm in a VERSAmax microplate reader (Molecular Devices, Sunnyvale, CA, USA). Viable cell percentage was calculated by defining cell viability in untreated samples as 100%.

#### Induction of adipocyte dysfunction

Mature adipocytes were incubated for 24, 48, 60, or 72 h with dexamethasone 0.1 μM or 1 mM^44,54^, either with or without insulin 1 μM. Some cells were cultured in the presence of 1 ng/mL of IL-1β^31,33,34^, for 24, 48, 60, or 72 h, either with or without insulin 1 μM, as an alternate method to induce adipocyte dysfunction. Different cellular density values were employed, depending on the assay to be performed.

#### Deoxyglucose consumption

To select the most efficient conditions to induce adipocyte dysfunction and evaluate the effectiveness of various concentrations of Aq-Cs SFs to control metabolic dysfunction, 2-DG consumption was determined with the Uptake 2-DG kit (Sigma MAK083), following the manufacturer’s instructions^55^. Briefly, the cells were washed twice with phosphate buffer saline (PBS) and incubated in FBS-free DMEM for 4 h. The cells were washed three more times and incubated for 40 min in KRPH buffer (HEPES 20 mM, KH_2_PO_4_ 5 mM, MgSO_4_ 1 mM, CaCl_2_ 1 mM, NaCl 136 mM, and KCl 4.7 mM) plus 2% BSA. Then, the cells were stimulated with insulin 1 μM for 20 min; 2-DG 10 mM was added, mixed, and incubated for 20 min. The cells were then lysed by a cold-heat cycle, and the assay buffer plus the enzyme mixture (Sigma, Cat. No. MAK083E) (8:2) was added; the plates were incubated at 37 °C for 60 min in the dark; then, extraction buffer was added and left to stand for 5 min, and the reaction was stopped by adding Reaction Mix B (53% glutathione reductase, 42% substrate-DTNB, and 5% recycling Mix). The plates were thoroughly mixed, and absorbance was measured at 412 nm.

#### Glycerol release

To select the optimal conditions to induce adipocyte dysfunction and evaluate the effectiveness of various concentrations of Aq-Cs SFs to revert dysfunction, glycerol concentration was quantified in the medium using the Colorimetric Assay Glycerol Kit (Sigma, Cat. No. MAK117)^56^. Briefly, 10 μL of culture medium for each treatment were transferred to 96-well plates, and 100 μL/well of the Master Reaction Mix was added; the plates were incubated for 20 min at RT, in the dark. Then, absorbance at 570 nm was measured.

#### IL-6 quantification in culture medium by sandwich ELISA

Adipocytes were treated for 24, 48, 60, or 72 h either with dexamethasone 1 μM or 1 ng/mL of IL-1β. After incubation, the culture medium was harvested, and IL-6 concentration was measured by ELISA (OptEIA^TM^ BD, 555240, San Diego, CA, USA), following the manufacturer’s instructions^26^. Briefly, flat-bottomed ELISA 96-well plates were covered with the capture antibody and incubated overnight at 4 °C in carbonate buffer 0.1 M (pH 9.6). Non-specific binding sites were blocked for 60 min at RT with 5% FBS in PBS. The samples were added, and the plates were incubated for 2 h at RT. Then, the plates were incubated with the HRP-conjugated detection anti-cytokine antibody for 60 min at RT. Tetramethylbenzidine was added as a substrate, and after 30 min of incubation at 37 °C, in the dark, the reaction was stopped with H_2_SO_4_ 2 N. The absorbance was determined at 450 nm in a VERSAmax ELISA plate reader. IL-6 concentration was calculated as pg/mg protein based on a standard curve. Protein content was determined using the Bradford assay according to the manufacturer’s instructions.

#### O_2_^•−^quantification

Superoxide anion (O_2_^•−^) was detected using dihydroethidium (DHE), which is oxidized to ethidium^57^. Briefly, adipocytes were cultured in 12-well plates and incubated either with or without 1 ng/mL of IL-1β and/or insulin 1 μM for 24, 48, 60, or 72 h. DHE 20 μM was added five min before incubation time was completed. Then, the plates were washed three times with PBS. After adding DMEM plus 10% FBS, the cells were photographed in a Cytation 5 cell image multimodal plate lector (Biotek Instruments, Winooski, VT, USA) under a 20X objective and analyzed with the Gen 5 software. Fluorescence intensity was quantified with the MetaMorph image analysis software v.6.1 (Molecular Devices, Sunnyvale, CA, USA).

#### 2-DG consumption and glycerol release response surface model

A 2-DG consumption and glycerol release response surface model was constructed to determine the most effective combinations of the SFs to control dysfunctional adipocytes and their concentrations^23^. In the experimental design, the effect of the variables *X*_1_, *X*_2_, and *X*_3_, which correspond to effective concentrations of SF1, SF2, and SF3, on two response variables, *Y*_1_ and *Y*_2_ (2-DG consumption and glycerol release) was evaluated. The concentrations of the Aq-Cs SFs that most efficiently increased 2-DG consumption were 40 μg/mL (SF1 and SF2) and 20 μg/mL (SF3), and the concentrations that most efficiently decreased glycerol release were 5 μg/mL (SF1 and SF3) and 40 μg/mL (SF2); therefore, these concentrations were used to define the three independent variables (Table 4).

**Table 4:** Experimental independent variables and levels used in this study.

	Variable	Levels			
		Symbols	Codified^a^		
			−1	0	1
Glycerol release	SF1	*X*_1_	2.5 μg/mL	5 μg/mL	7.5 μg/mL
	SF2	*X*_2_	20 μg/mL	40 μg/mL	60 μg/mL
	SF3	*X*_3_	2.5 μg/mL	5 μg/mL	7.5 μg/mL
2-DG consumption	SF1	*X*_1_	20 μg/mL	40 μg/mL	60 μg/mL
	SF2	*X*_2_	20 μg/mL	40 μg/mL	60 μg/mL
	SF3	*X*_3_	10 μg/mL	20 μg/mL	30 μg/mL

^a^Coded levels for the independent variables: maximum (1), intermediate (0), and minimum (−1). The amplitude of the outer levels was 50%.

A factorial design was adjusted to 2^3-1^ quadratic polynomial models with five combinations in total (M1-M5). The variables were coded according to the following equation:

1 $${x}_{i}=({X}_{l}-{X}_{0})/\Delta {X}_{I}$$

where *x*_*i*_ is the codified value for the independent variable; *X*_*i*_ is the actual value of the independent variable; *X*_0_ is the value of the independent variable at the central point, and Δ*x*_*i*_ is the incremental value of the independent variable.

The predictive model for the optimal point was expressed according to the following Eq. (2):

2 $${Y}_{n}=bo+\mathop{\sum }\limits_{i=1}^{3}\,{b}_{i}{X}_{i}+\mathop{\sum }\limits_{i=1}^{3}\,{b}_{ii}{X}_{i}^{2}+\mathop{\sum }\limits_{i < j=1}^{3}\,{b}_{ij}{X}_{i}{X}_{j}$$

where *Y*_*n*_ are the response variables, *b*_0_ is the regression coefficient, and *X*_*i*_ is the coded level of each independent variable. Data were analyzed by applying the regression method for the response surface, using the software Minitab® v18.1. Three levels were coded for the independent variables: maximum, intermediate, and minimum, where the amplitude of the outer levels was 50%.

#### Statistical analysis

All measured parameters were compiled in Excel. Differences were tested by ANOVA and the post-hoc Tukey-Kramer test. Data were analyzed with the INSTAT 3 GraphPad software by uni- and multi-varied analyses. P ≤ 0.05 was regarded as statistically significant.

## Supplementary information

Supplementary Figures

## Data Availability

The material is held by the authors.

## References

[1] Blancas FG (2010). Obesity as an inflammatory process. Bol. Med. Hosp. Infant..

[2] Hernández, M. Encuesta Nacional de Salud y Nutrición de Medio Camino 2016: Resultados ponderados, https://www.gob.mx/salud/documentos/encuesta-nacional-de-salud-y-nutricion-de-medio-camino-2016 (2016).

[3] Kaur Jaspinder (2014). A Comprehensive Review on Metabolic Syndrome. Cardiology Research and Practice.

[4] Park Jinbong, Jeon Yong-Deok, Kim Hye-Lin, Lim Hara, Jung Yunu, Youn Dong-Hyun, Jeong Mi-Young, Kim Hyun-Ju, Kim Sung-Hoon, Kim Su-Jin, Hong Seung-Heon, Um Jae-Young (2013). Interaction ofVeratrum nigrumwithPanax ginsengagainst Obesity: A Sang-ban Relationship. Evidence-Based Complementary and Alternative Medicine.

[5] Manabe I (2011). Chronic inflammation links cardiovascular, metabolic and renal diseases. Circ. J..

[6] Ahima RS (2006). Adipose tissue as an endocrine organ. Obesity (Silver Spring)..

[7] Grundy SM (2004). What is the contribution of obesity to the metabolic sindrome?. Endrocrinol. Metab. Clin. North Am..

[8] Alberti KG, Zimmet P, Shaw J (2006). Metabolic syndrome-a new world-wide definition. A Consensus statement from the international Diabetes Federation. Diabet. Med..

[9] Martyn JA, Kaneki M, Yasuhara S (2008). Obesity-Induced Insulin Resistance and Hyperglycemia: Etiological Factors and Molecular Mechanisms. Anesthesiology.

[10] Krebs M, Roden M (2005). Molecular mechanisms of lipid‐induced insulin resistance in muscle, liver and vasculature. Diabetes, Obesity and Metabolism..

[11] Hirabara Sandro M., Gorjão Renata, Vinolo Marco A., Rodrigues Alice C., Nachbar Renato T., Curi Rui (2012). Molecular Targets Related to Inflammation and Insulin Resistance and Potential Interventions. Journal of Biomedicine and Biotechnology.

[12] He Yuting, Zhang Ling, Zhu Zhuoli, Xiao Anqi, Yu Haiyang, Gan Xueqi (2017). Blockade of cyclophilin D rescues dexamethasone-induced oxidative stress in gingival tissue. PLOS ONE.

[13] Schönfeld P, Wojtczak L (2008). Fatty acids as modulators of the cellular production of reactive oxygen species. Free Radic. Biol. Med..

[14] Murdolo G (2013). Oxidative stress and lipid peroxidation by-products at the crossroad between adipose organ dysregulation and obesity-linked insulin resistance. Biochimie..

[15] Yao Longbiao, Herlea-Pana Oana, Heuser-Baker Janet, Chen Yitong, Barlic-Dicen Jana (2014). Roles of the Chemokine System in Development of Obesity, Insulin Resistance, and Cardiovascular Disease. Journal of Immunology Research.

[16] Chen H (2006). Cellular inflammatory responses: Novel insights for obesity and insulin resistance. Pharmacol. Res..

[17] Naik VR, Agshikar NV, Abraham GJ (1980). Analgesic and anti-inflammatory activity in alcoholic extracts of *Cucumis trigonus* Roxburghii. A preliminary communication. Pharmacology..

[18] Naito Y (2005). Reduction of diabetes-induced renal oxidative stress by a cantaloupe melon extract/gliadin biopolymers, oxykine, in mice. Biofactors..

[19] Veeramani C, Aristatle B, Pushpavalli G, Pugalendi KV (2010). Antihypertensive efficacy of *Melothria maderaspatana* leaf extract on sham-operated and uninephrectomized DOCA-salt hypertensive rats. J. Basic Clin. Physiol. Pharmacol..

[20] Vouldoukis I (2004). Antioxidant and anti-inflammatory properties of a *Cucumis melo* LC. extract rich in superoxide dismutase activity. J. Ethnopharmacol..

[21] Zuhair HA, Abd El-Fattah AA, El-Sayed MI (2000). Pumpkin-seed oil modulates the effect of felodipine and captopril in spontaneously hypertensive rats. Pharmacol Res..

[22] Soltani R (2017). Evaluation of the Effects of *Cucumis sativus* Seed Extract on Serum Lipids in Adult Hyper lipidemic Patients: A Randomized Double-Blind Placebo-Controlled Clinical Trial. J. Food Sci..

[23] Paseephol T, Small D, Sherkat F (2007). Process optimization for fractionating Jerusalem artichoke fructans with ethanol using response surface methodology. Food Chem..

[24] Sakoda H (2000). Dexamethasone-Induced Insulin Resistance in 3T3-L1 Adipocytes Is Due to Inhibition of Glucose Transport Rather Than Insulin Signal Transduction. Diabetes..

[25] Surai PF (2015). Silymarin as a natural antioxidant: an overview of the current evidence and perspectives. Antioxidants..

[26] Trejo-Moreno Celeste, Méndez-Martínez Marisol, Zamilpa Alejandro, Jiménez-Ferrer Enrique, Perez-Garcia Maria, Medina-Campos Omar, Pedraza-Chaverri José, Santana María, Esquivel-Guadarrama Fernando, Castillo Aida, Cervantes-Torres Jacquelynne, Fragoso Gladis, Rosas-Salgado Gabriela (2018). Cucumis sativus Aqueous Fraction Inhibits Angiotensin II-Induced Inflammation and Oxidative Stress In Vitro. Nutrients.

[27] He J (2015). Thiazolidinediones attenuate lipolysis and ameliorate dexamethasone-induced insulin resistance. Metabolism..

[28] Ottens Thomas H, Nijsten Maarten, Hofland Jan, Dieleman Jan M, Hoekstra Miriam, van Dijk Diederik, van der Maaten Joost (2015). Effect of high-dose dexamethasone on perioperative lactate levels and glucose control: a randomized controlled trial. Critical Care.

[29] Xu C, Xu GH (2009). Glucocorticoids, adipose metabolism and insulin resistance. Sheng Li Ke Xue Jin Zhan..

[30] Wong RHF, Sul HS (2010). Insulin signaling in fatty acid and fat synthesis: a transcriptional perspective. Curr. Opin. Pharmacol..

[31] Kagoshima M, Ito K, Cosio B, Adcock IM (2003). Glucocorticoid suppression of nuclear factor-κB: a role for histone modifications. Bioche. Soc. Trans..

[32] Arner P, Langin D (2014). Lipolysis in lipid turnover, cancer cachexia, and obesity-induced insulin resistance. Trends Endocrinol. Metab..

[33] Rotter V, Nagaev I, Smith U (2003). Interleukin-6 (IL-6) induces insulin resistance in 3T3-L1 adipocytes and is, like IL-8 and tumor necrosis factor-alpha, overexpressed in human fat cells from insulin-resistant subjects. J. Biol. Chem..

[34] Hsieh Chia-Chien, Chou Mei-Jia, Wang Chih-Hsuan (2017). Lunasin attenuates obesity-related inflammation in RAW264.7 cells and 3T3-L1 adipocytes by inhibiting inflammatory cytokine production. PLOS ONE.

[35] Song MJ, Kim KH, Yoon JM, Kim J (2006). B Activation of Toll-like receptor 4 is associated with insulin resistance in adipocytes. Biochem. Biophys. Res. Commun..

[36] Erusan R, Nalini D, Manohar G, Malathi R (2012). Correlation between obesity and inflammation in cardiovascular diseases—evaluation of leptin and inflammatory cytokines. Open J. Endocr. Metabol. Dis..

[37] Yan-Do R, MacDonald PE (2017). Impaired “Glycine”-mia in Type 2 Diabetes and Potential Mechanisms Contributing to Glucose Homeostasis. Endocrinology..

[38] Jaganjac M (2017). Combined metformin and insulin treatment reverses metabolically impaired omental adipogenesis and accumulation of 4-hydroxynonenal in obese diabetic patients. Redox Biol..

[39] Alarcon AFJ (2008). Glycine regulates the production of pro-inflammatory cytokines in lean and monosodium glutamate-obese mice. Eur J Pharmacol..

[40] Reyes YL (2016). Effect of Glycine on Adipocyte Hypertrophy in a Metabolic Syndrome Rat Model. Curr. Drug. Deliv..

[41] Bogdanski P (2013). Supplementation with L-arginine favorably influences plasminogen activator inhibitor type 1 concentration in obese patients. A randomized, double blind trial. J Endocrinol Invest..

[42] Fu WJ (2005). Dietary L-Arginine Supplementation Reduces Fat Mass in Zucker Diabetic Fatty Rats. Nutr..

[43] Zambrano ML, Rodríguez DB, Álvarez A (2007). Kinetic Study and Surface Response Analysis on the Rehydration of Frozen-dried Carrot (*Daucus carota*). Información Tecnológica..

[44] Klein HH (2002). Differential modulation of insulin actions by dexamethasone: Studies in primary cultures of adult rat hepatocytes. J Hepatol..

[45] Manna SK, Mukhopadhyay A, Van NT, Aggarwal BB (1999). Silymarin Suppresses TNF-Induced Activation of NF-κB, c-Jun N-Terminal Kinase, and Apoptosis. J. Immunol..

[46] Shafiei-Roudbari Seyedeh-Khadijeh, Malekinejad Hassan, Janbaz-Aciabar Hamed, Razi Mazdak (2017). Crosstalk between E2F1 and P53 transcription factors in doxorubicin-induced DNA damage: evidence for preventive/protective effects of silymarin. Journal of Pharmacy and Pharmacology.

[47] Almanza-Perez JC (2010). Glycine regulates inflammatory markers modifying the energetic balance through PPAR and UCP-2. Biomed. Pharmacother..

[48] Ruiz-Ramírez A, Ortiz-Balderas E, Cardozo-Saldaña G, Diaz-Diaz E, El-Hafidi M (2013). Glycine restores glutathione and protects against oxidative stress in vascular tissue from sucrose-fed rats. Clin. Sci..

[49] Fortis BA (2013). *Cucurbita ficifolia* Bouché (Cucurbitaceae) and D-chiro-inositol modulate the redox state and inflammation in 3T3-L1 adipocytes. J. Pharm. Pharmacol..

[50] Stolf AM (2018). Effects of silymarin on angiogenesis and oxidative stress in streptozotocin-induced diabetes in mice. Biomed. Pharmacother..

[51] Lovelace ES (2015). Silymarin Suppresses Cellular Inflammation By Inducing Reparative Stress Signaling. J. Nat. Prod..

[52] Patanè G (2000). Metformin restores insulin secretion altered by chronic exposure to free fatty acids or high glucose: a direct metformin effect on pancreatic beta-cells. Diabetes..

[53] Stumvoll M, Nurjhan N, Perriello G, Dailey G, Gerich JEN (1995). Metabolic effects of metformin in non-insulin-dependent diabetes mellitus. Engl J Med..

[54] Kajita K (2001). Glucocorticoid-Induced insulin resistance associates with activation of protein kinase C isoforms. Cell. Signal..

[55] Yamamoto N (2010). An enzymatic fluorimetric assay to quantitate 2-deoxyglucose and 2-deoxyglucose-6-phosphate for *in vitro* and *in vivo* use. Anal Biochem..

[56] Kim GS (2012). *Citrus aurantium* flavonoids inhibit adipogenesis through the Akt signaling pathway in 3T3-L1 cells. BMC Complement Altern Med..

[57] Pedraza CJ (2009). ROS scavenging capacity and neuroprotective effect of alpha-mangostin against 3-nitropropionic acid in cerebellar granule neurons. Exp. Toxicol. Pathol..

