# Supplementary material for: Effect of Cucumis sativus on Dysfunctional 3T3-L1 Adipocytes
Source: Sci Rep. 2019 Sep 16;9:13372. doi: 10.1038/s41598-019-49458-6 (PMC6746747; doi:10.1038/s41598-019-49458-6)
Supplement: Supplementary file 1 — Supplementary Figures [file 41598_2019_49458_MOESM1_ESM.pdf]

Supplementary Material

Article in Scientific Reports

EFFECT OF Cucumis sativus ON DYSFUNCTIONAL 3T3-L1 ADIPOCYTES

Méndez-Martínez Marisol, Trejo-Moreno Celeste, Maldonado-Mejía Laura, Esquivel-Guadarrama Fernando, Pedraza-Chaverri José, Zamilpa Alejandro, Medina-Campos Omar, Alarcón-Aguilar Francisco, Almanza-Pérez Julio, Contreras-Nuñez Erika, Santana-Calderón Angélica, Fragoso Gladis, Jiménez-Ferrer Enrique, and Rosas Gabriela

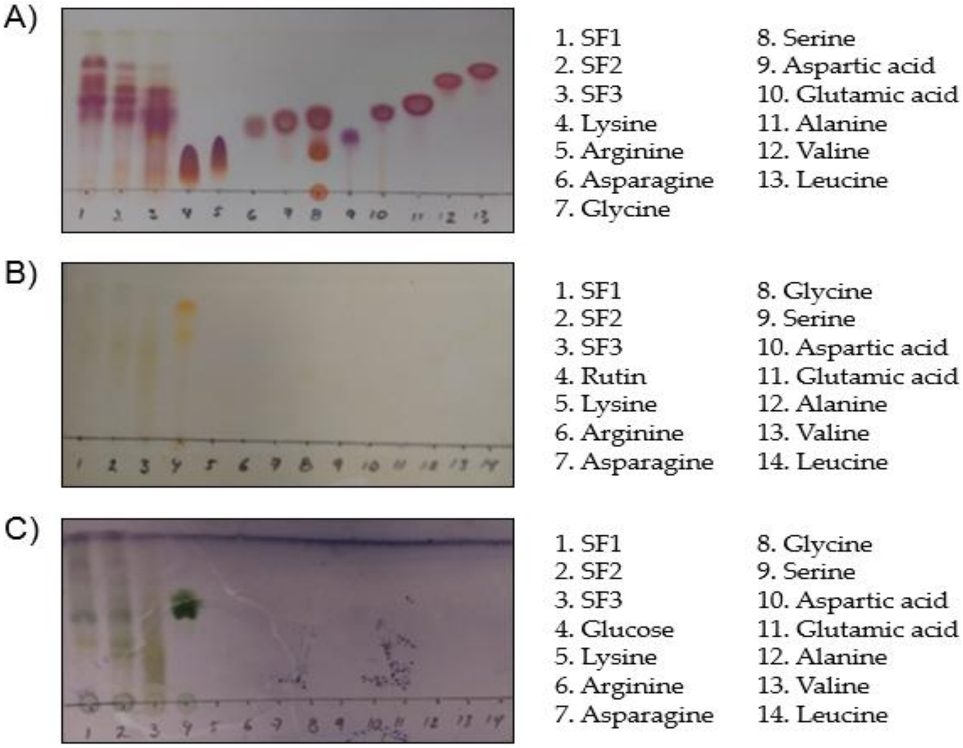

Supplementary Figure 1. Thin-layer chromatography. Separation of the different compounds of the subfraction of *Cucumis sativus* using normal phase TLC and the system n-butanol: acetone: glacial acetic acid: water (35:35:10:20 v/v). The plates were revealed with ninhydrin to visualize amino acids (A), with aminoethanol dimethylborate to flavonoids (B), and 4-hydroxybenzaldehyde to polisaccharides (C).

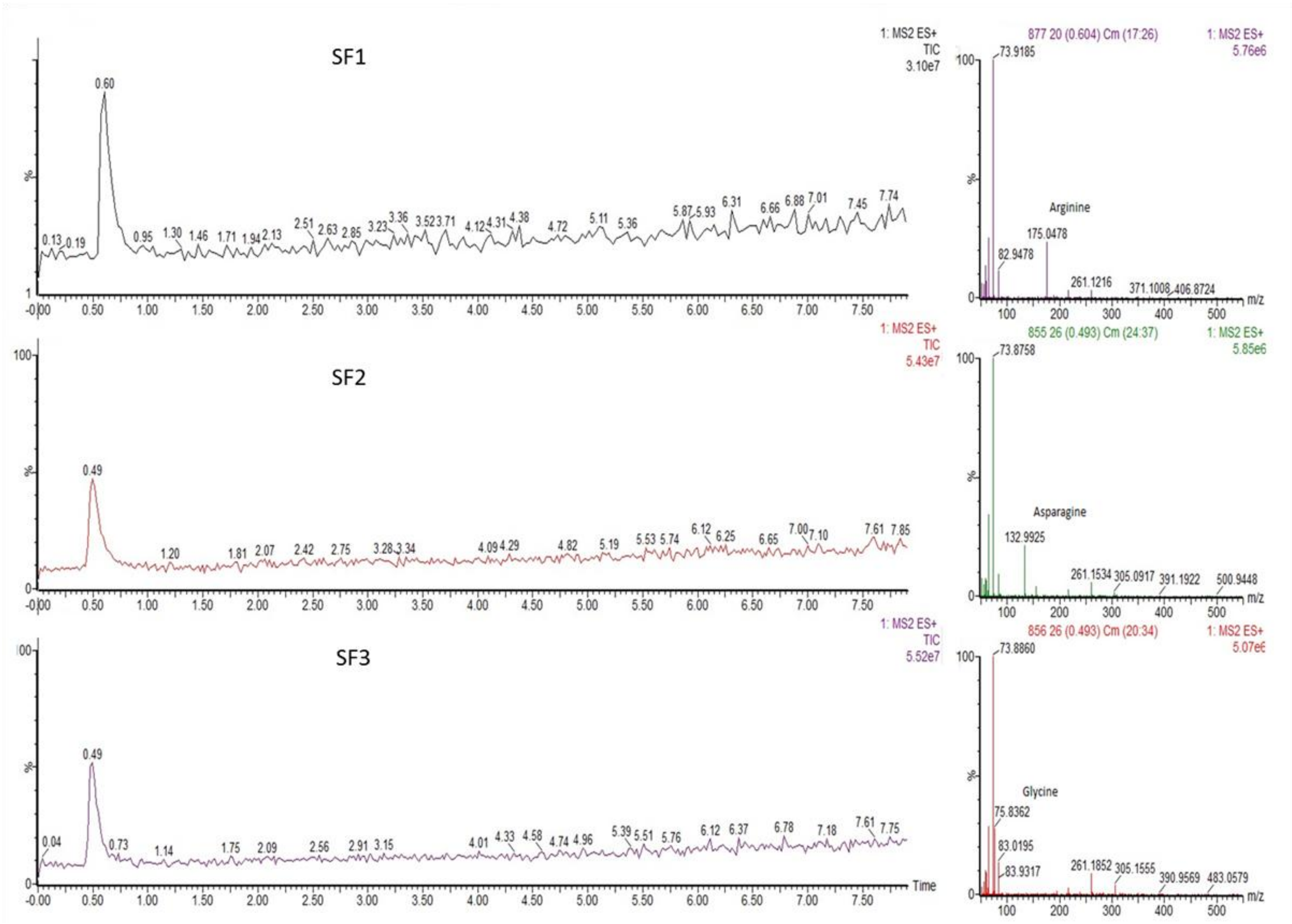

Supplementary Figure 2. Chromatograms of SF1, SF2 and SF3 fractions and for Arginine (175.0478), Asparagine (132.9925), and Glycine (75.8362) amino acids.

A)

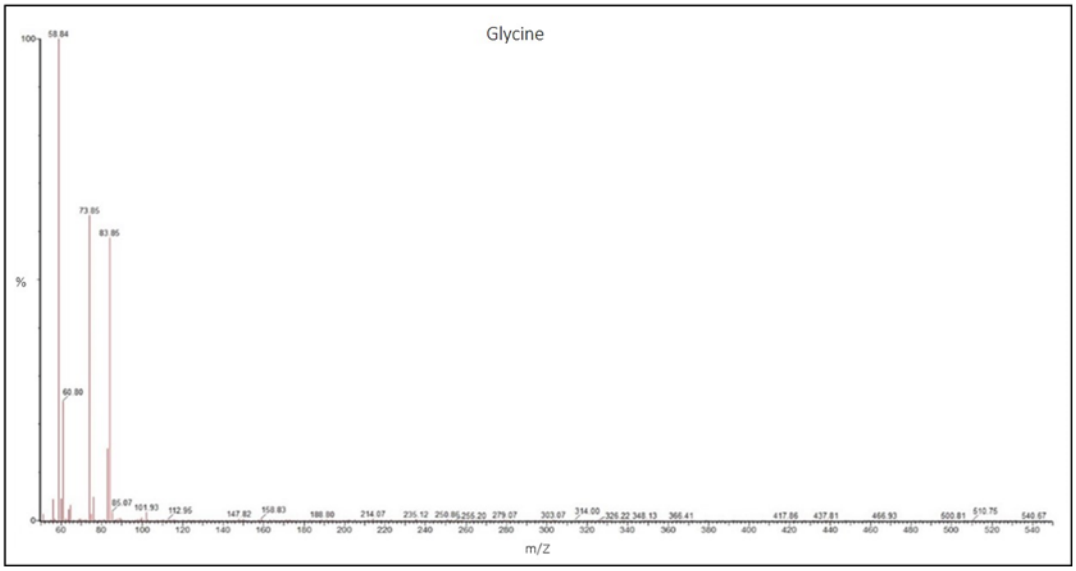
Glycine
100
%
0
58.84
73.85
83.85
60.80
m/Z

B)

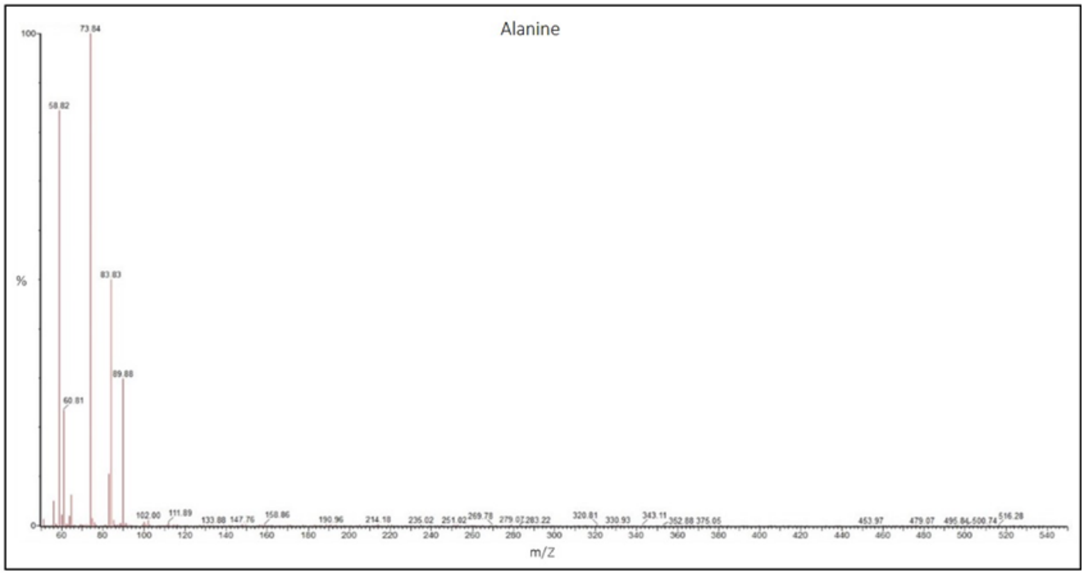
Alanine
100
%
0
73.84
58.82
83.83
89.88
60.81
m/Z

C)

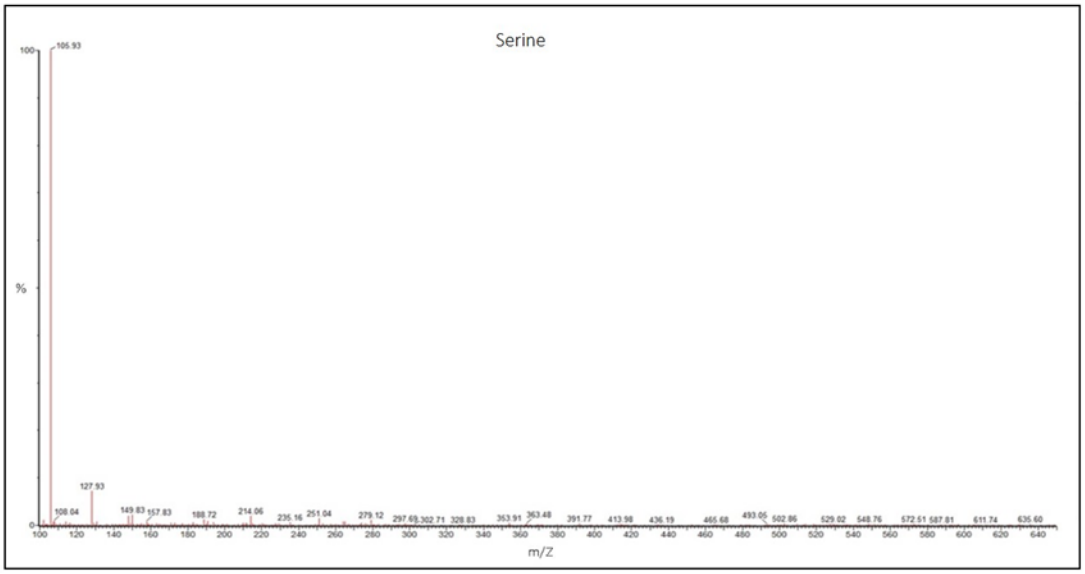
Serine
100
%
0
105.93
127.93
m/Z

D)

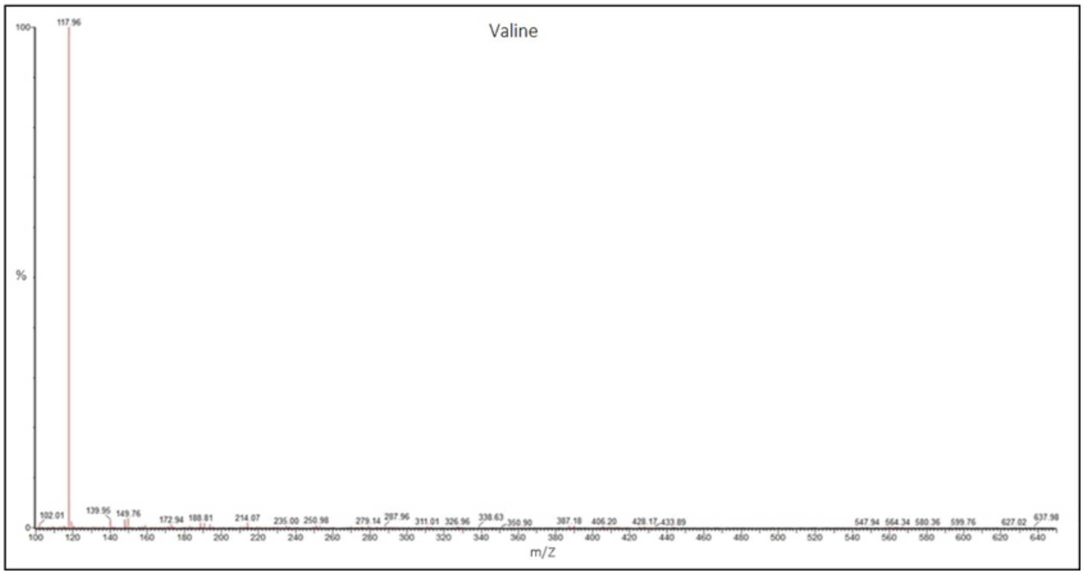

E)

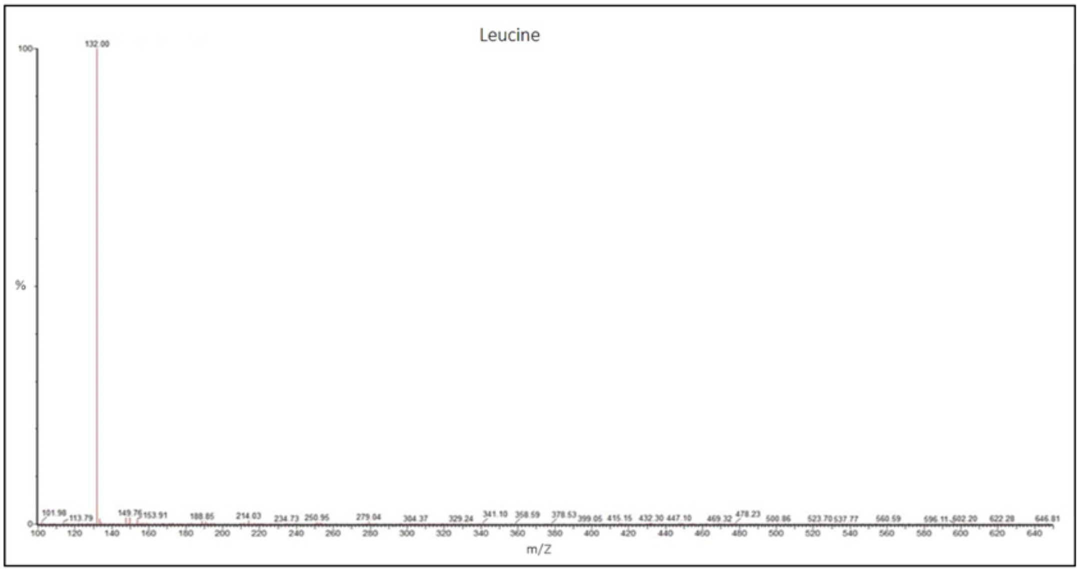

F)

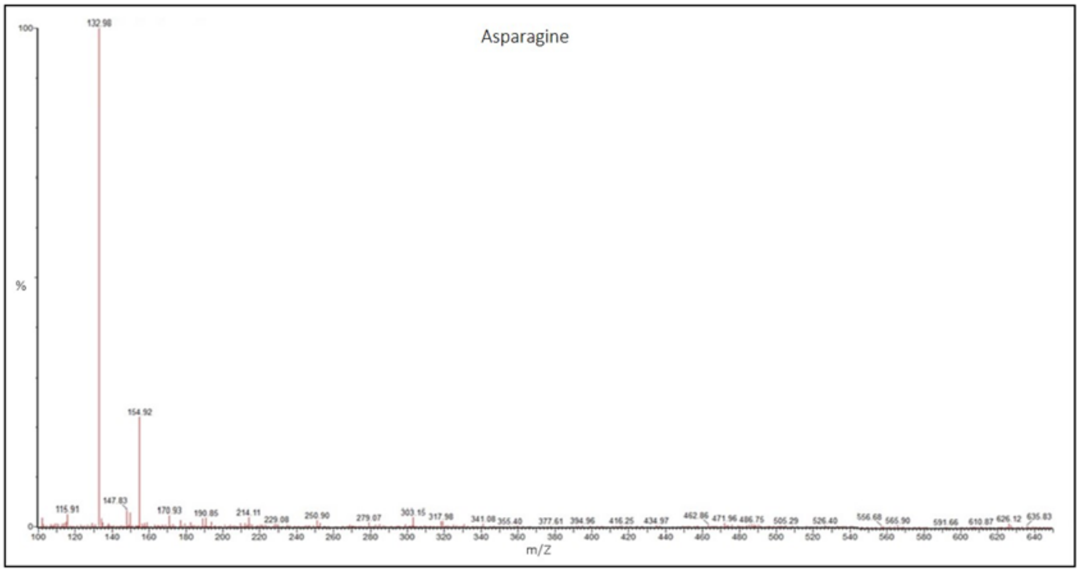

G)

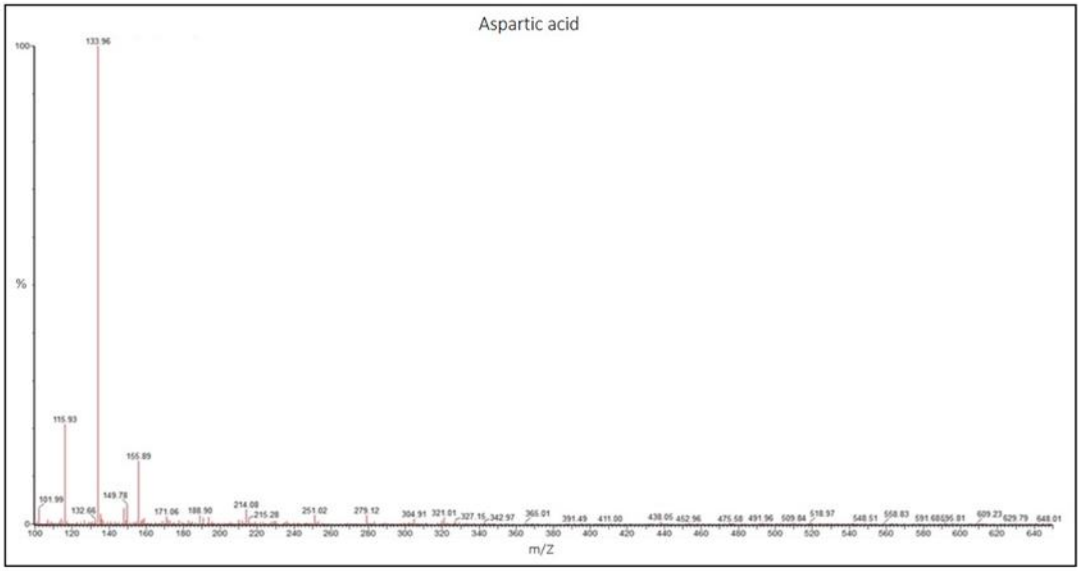
Aspartic acid
133.96
115.93
155.89
%
m/Z

H)

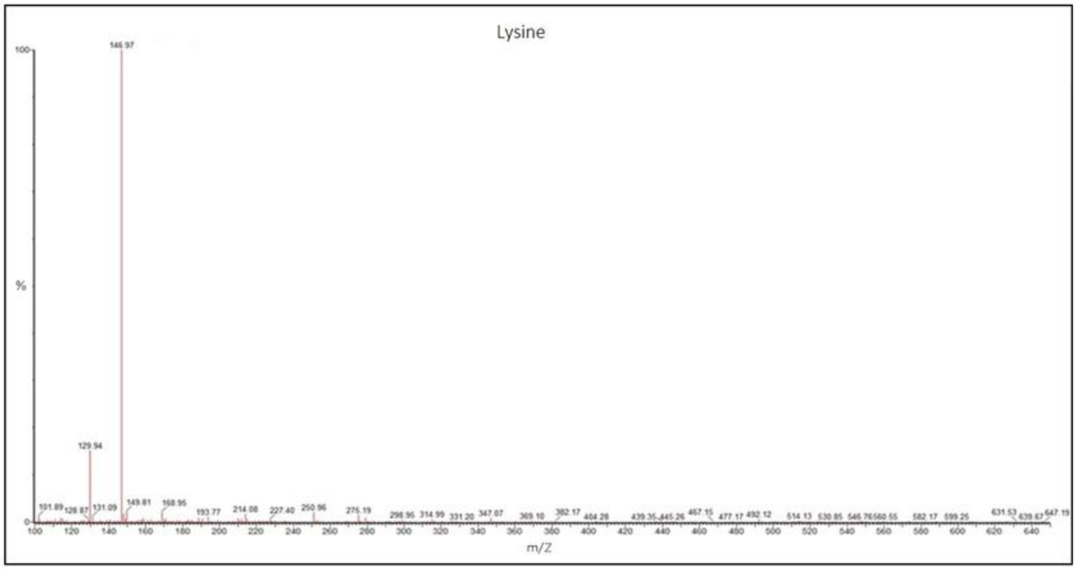
Lysine
146.97
129.94
%
m/Z

I)

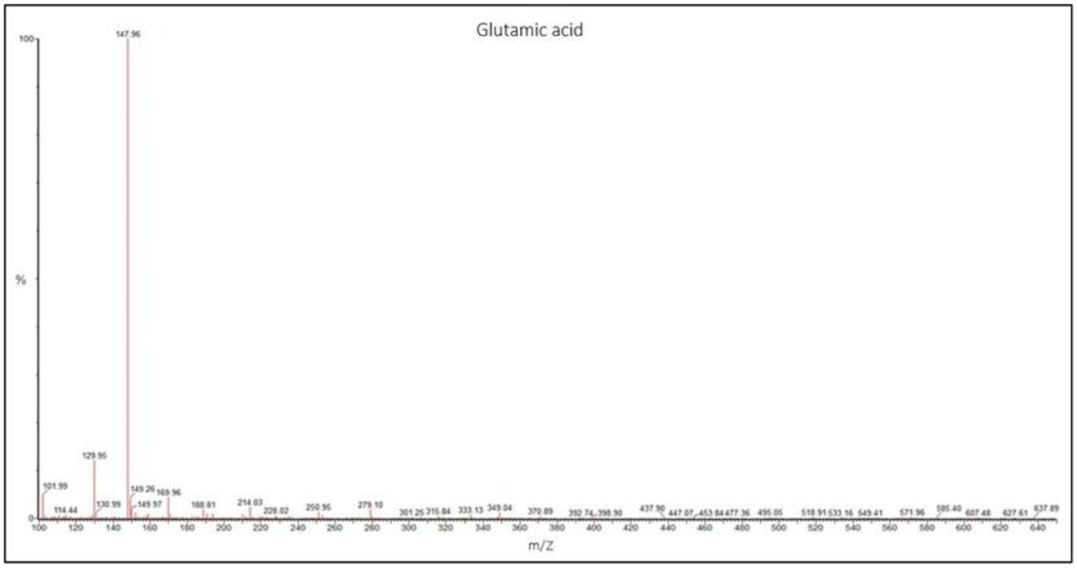
Glutamic acid
147.96
129.95
%
m/Z

J)

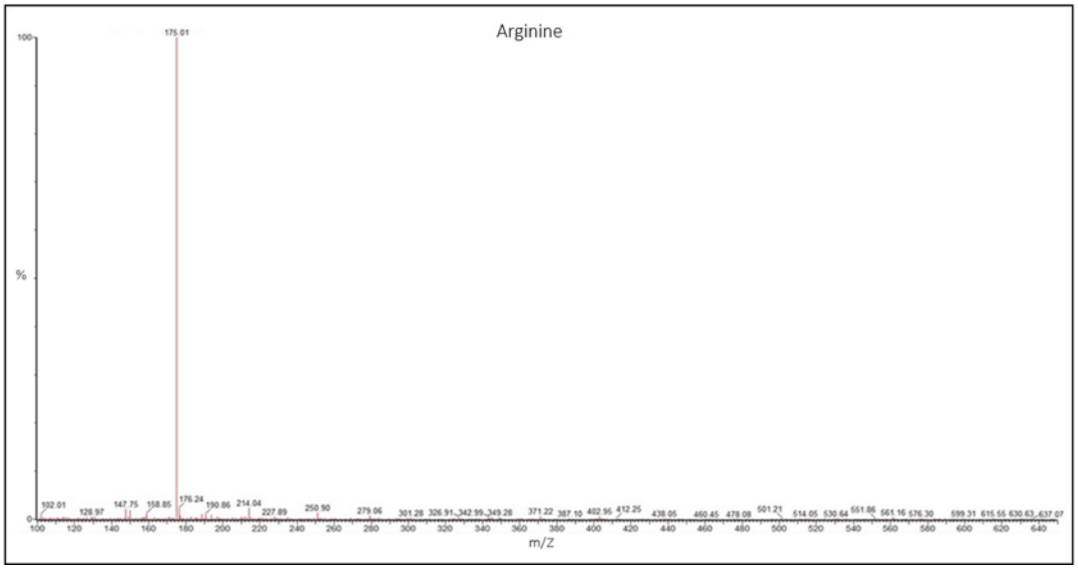

Supplementary Figure 3. Mass spectrometry Glycine (A), Alanine (B), Serine (C) Valine (D), Leucine (E), Aspartic acid (F), Asparagine (G), Lysine (H), Glutamic acid (I), and Arginine (J).
